# Respuesta del autor a la carta al editor titulada Reflexiones sobre la profilaxis de los arbovirus en América Latina

**DOI:** 10.26633/RPSP.2019.82

**Published:** 2019-09-30

**Authors:** Viviana Masciadri

**Affiliations:** 1 Consejo Nacional de Investigaciones Científicas y Técnicas Consejo Nacional de Investigaciones Científicas y Técnicas Buenos Aires Argentina Consejo Nacional de Investigaciones Científicas y Técnicas, Buenos Aires, Argentina.

Al editor:

Aunque en nuestro artículo original (1) asumimos que la tasa de incidencia de dengue es y será el indicador que mejor mide los aciertos y desaciertos en la voluntad política de los gobiernos, la coordinación intersectorial, la participación de la comunidad y el fortalecimiento de las leyes sanitarias nacionales para el control del dengue, esta no es el único factor que debe considerarse.

En efecto, se señala que existe una vasta bibliografía que ayuda a comprender los determinantes sociales, ambientales y climáticos tanto en el medio urbano como en los ecosistemas vinculados a las áreas de frontera colindantes con espacios navegables o terrestres. También se conoce la asociación de la transmisión de enfermedades ligadas al vector con el tránsito de viajeros y la movilidad de la población que habita áreas de frontera entre los estados miembros y asociados al Mercosur. El factor comercial ligado a la comercialización de neumáticos usados y otros productos en el Mercosur es un determinante menos indagado, aunque Chile adoptó medidas comerciales explícitas desde la óptica sanitaria. La inequidad que representa la proliferación de las enfermedades desatendidas y la resistencia a los avances provenientes del campo de la investigación básica (2) podrían estar incidiendo en la falta de coordinación a escala subregional y hemisférica (Mercosur, Pacto Andino, Mercado Común Centroamericano y Tratado de libre comercio de América del Norte) (3) impidiendo la conformación de fondos que apoyen la contención de las enfermedades trasmitidas por arbovirus.

En cuanto a las nuevas tecnologías se destaca el aporte clave de la Plataforma de Información en Salud de las Américas (PLISA), que permite visibilizar el aspecto nacional, subregional y hemisférico de la incidencia de dengue. En el último cuatrienio, si se comparan los años en los cuales la incidencia fue superior a 50 casos de dengue por cien mil habitantes, se observa que en la Región de las Américas se registraron 224,98 casos en 2016 y 210,29 casos en 2019 por cien mil habitantes; en los Estados Miembros del Mercosur se registraron 559,88 casos de dengue por cien mil habitantes en el año 2016 y 576,24 en el 2019; en los Estados Asociados al Mercosur la cifra fue de 126,30 en 2016 y 73,78 en 2019 por cien mil habitantes.

En 2016, entre los Estados Miembros del Mercosur se destacan Paraguay (1 044,09) y Brasil (716,01), y entre los Estados Asociados, Colombia (207,62).

En 2019, el ranking epidemiológico ha cambiado; las tasas de incidencia de dengue por cien mil habitantes son de 838,62 en Brasil (SE28), 151,06 en Colombia (SE31) y 120,91 en Paraguay (SE31).

En relación con el aporte de las nuevas tecnologías se enfatiza el valor de las alertas epidemiológicas tempranas como la recientemente emitida en Argentina (4,5), que deben trascender los portales de los Sistemas Nacionales de Vigilancia Sanitaria y alcanzar a vastos sectores de la sociedad: al sector salud (primer, segundo y tercer nivel de atención) propiamente dicho; a espacios de tránsito de personas (aeropuertos, puertos, trenes, buses, subterráneos); a escuelas, colegios secundarios y universidades; a sitios donde se comercializan productos que favorecen la proliferación del vector (neumáticos usados, bambú de la suerte, floreros para cementerios, etc.); a clubes de barrio y a los medios masivos de comunicación y a los partidos políticos.

En síntesis, para controlar al vector en las áreas urbanas o rurales, las organizaciones gubernamentales y no gubernamentales comprometidas con la sociedad podrían colaborar en campañas que multipliquen la información. La proliferación de las enfermedades transmitidas por arbovirus adquiere dimensiones transfronterizas acarreando los estigmas de la marginalidad, la migración y la pobreza (6, 7). Una campaña que deconstruya prejuicios y manifieste qué #NingúnMosquitoDiscrimina# resulta imprescindible.

## Declaración.

Las opiniones expresadas en este manuscrito son responsabilidad de los autores y no reflejan necesariamente los criterios ni la política de la RPSP/PAJPH y/o de la OPS.
